# Ammonia is associated with liver-related complications and predicts mortality in acute-on-chronic liver failure patients

**DOI:** 10.1038/s41598-024-56401-x

**Published:** 2024-03-09

**Authors:** Kessarin Thanapirom, Sombat Treeprasertsuk, Ashok Choudhury, Nipun Verma, Radha Krishan Dhiman, Mamun Al Mahtab, Harshad Devarbhavi, Akash Shukla, Saeed Sadiq Hamid, Wasim Jafri, Soek Siam Tan, Guan H. Lee, Hasmik Ghazinyan, Ajit Sood, Dong Joon Kim, C. E. Eapen, Han Tao, Nan Yuemin, A. Kadir Dokmeci, Manoj Sahu, Anil Arora, Ashish Kumar, Ramesh Kumar, V. G. Mohan Prasad, Ananta Shresta, Jose Sollano, Diana Alcantara Payawal, George Lau, Shiv Kumar Sarin

**Affiliations:** 1 Division of Gastroenterology, Faculty of Medicine, Chulalongkorn University and King Chulalongkorn Memorial Hospital, Thai Red Cross Society, Bangkok, Thailand; 2 Center of Excellence in Hepatic Fibrosis and Cirrhosis, Chulalongkorn University, Bangkok, Thailand; 3 Department of Hepatology, Institute of Liver and Biliary Sciences, New Delhi, India; 4 Department of Hepatology, Postgraduate Institute of Medical Education and Research, Chandigarh, India; 5 Department of Hepatology, Sanjay Gandhi Postgraduate Institute of Medical Sciences, Lucknow, Uttar Pradesh India; 6 Department of Hepatology, Bangabandhu Sheikh Mujib Medical University, Dhaka, Bangladesh; 7 Department of Hepatology, St John Medical College, Bangalore, India; 8 Department of Gastroenterology, Lokmanya Tilak Municipal General Hospital, and Lokmanya Tilak Municipal Medical College, Sion, Mumbai, India; 9 Department of Medicine, Aga Khan University Hospital, Karachi, Pakistan; 10 Department of Hepatology, Hospital Selayang, Bata Caves, Selangor Malaysia; 11 Yong Loo Lin School of Medicine, National University of Singapore, Singapore, Singapore; 12 Department of Hepatology, Nork Clinical Hospital of Infectious Disease, Yerevan, Armenia; 13 Department of Gastroenterology, DMC, Ludhiana, India; 14 Department of Internal Medicine, Hallym University College of Medicine, Seoul, South Korea; 15 Department of Hepatology, CMC, Vellore, India; 16 Department of Hepatology and Gastroenterology, The Third Central Clinical College of Tianjin Medical University, No. 83, Jintang Road, Hedong District, Tianjin, 300170 China; 17 Department of Traditional and Western Medical Hepatology, Third Hospital of Hebei Medical University, Shijiazhuang, China; 18 Department of Medicine, Ankara University School of Medicine, Ankara, Turkey; 19 Department of Gastroenterology and Hepatology Sciences, IMS and SUM Hospital, Bhubaneswar, Odisha India; 20 Institute of Liver Gastroenterology and Pancreatico Biliary Sciences, Sir Ganga Ram Hospital, New Delhi, India; 21 Department of Gastroenterology, All India Institute of Medical Sciences, Patna, Bihar India; 22 Department of Gastroenterology, VGM Hospital, Coimbatore, India; 23 Department of Hepatology, Alka Hospital Pvt Ltd, Jawalakhel, Lalitpur, Nepal; 24 Department of Medicine, Cardinal Santos Medical Center, Manila, Philippines; 25 Fatima University Medical Center Manila, Manila, Philippines; 26 Department of Medicine, Humanity, and Health Medical Group, Hong Kong, People’s Republic of China; 27 Senior Department of Hepatology, Fifth Medical Center of Chinese PLA General Hospital, Beijing, 100039 China; 28 Division of Gastroenterology, Department of Medicine, Faculty of Medicine, Chulalongkorn University, Bangkok, 10330 Thailand

**Keywords:** Hepatology, Biological techniques, Molecular medicine, Pathogenesis

## Abstract

The relationship between ammonia and liver-related complications (LRCs) in acute-on-chronic liver failure (ACLF) patients is not clearly established. This study aimed to evaluate the association between ammonia levels and LRCs in patients with ACLF. The study also evaluated the ability of ammonia in predicting mortality and progression of LRCs. The study prospectively recruited ACLF patients based on the APASL definition from the ACLF Research Consortium (AARC) from 2009 to 2019. LRCs were a composite endpoint of bacterial infection, overt hepatic encephalopathy (HE), and ascites. A total of 3871 cases were screened. Of these, 701 ACLF patients were enrolled. Patients with LRCs had significantly higher ammonia levels than those without. Ammonia was significantly higher in patients with overt HE and ascites, but not in those with bacterial infection. Multivariate analysis found that ammonia was associated with LRCs. Additionally, baseline arterial ammonia was an independent predictor of 30-day mortality, but it was not associated with the development of new LRCs within 30 days. In summary, baseline arterial ammonia levels are associated with 30-day mortality and LRCs, mainly overt HE and ascites in ACLF patients.

## Introduction

Hyperammonemia is a major contributor to hepatic encephalopathy (HE) development^1–3^. The cerebral effect of ammonia is linked to systemic inflammation and bacterial translocation^4^. In patients with acute liver failure (ALF), hyperammonemia is correlated with brain edema and herniation^5^. However, this relationship is less well demonstrated in cirrhosis^2,6,7^. In ACLF patients with HE, intracranial hypertension, cerebral edema, and herniation are uncommon. Key factors associated with the presence and severity of HE are ammonia, abnormal brain oxygen consumption, and systemic inflammation. Improvements in these factors are associated with lower HE grades in ACLF patients^8^.

Ammonia metabolism is a multiorgan process that involves the liver, brain, kidneys, muscle, and gastrointestinal systems. As a result, hyperammonemia may indicate poor non-cerebral function reserves, ongoing multiorgan impairment, and malnutrition^9^. Several recent studies have highlighted the association between hyperammonemia and poor outcomes, including liver-related complications (LRCs), hospitalization, progression to ACLF, and mortality in patients with clinically stable advanced chronic liver disease or cirrhosis^10,11^. Previous studies revealed that ammonia was closely related to ACLF grade, organ failure in the liver, kidney, and brain, and is an independent risk factor for 28-day mortality^12,13^. These findings provided evidence that ammonia levels not only have clinical usefulness in evaluating HE severity but may also be a potential prognostic biomarker for identifying patients at high risk of poor outcomes. The relationship between plasma ammonia levels and other LRCs, including bacterial infection, ascites, and 30-day mortality in ACLF, is less well defined. Therefore, this study aimed to investigate the association between ammonia levels and LRCs in patients with ACLF and the efficacy of ammonia levels in predicting mortality and the development of new LRCs during the 30 days after hospitalization.

## Methods

### Study design and participants

This study prospectively collected data from the Asian Pacific Association for the Study of the Liver (APASL)-ACLF Research Consortium (AARC) database, which included hospitalized ACLF patients from 31 centers between April 2009 and December 2019. Inclusion criteria were hospitalized patients with ACLF. Exclusion criteria were: age < 18 years, death within 24 h after admission, acute liver failure, pregnancy, human immunodeficiency virus infection, hepatocellular carcinoma, extrahepatic malignancy, or subjects missing key parameters such as ammonia levels. Patient characteristics, ACLF grade, organ failure events, and LRCs were recorded at baseline until day 30 or death. The diagnosis of ACLF was based on the APASL criteria, and grading of ACLF was determined by the AARC score^14^. ACLF grade I, II, and III was determined if the AARC was 5–7, 8–10, and 11–15, respectively^14^.

LRCs were defined as having at least one decompensating event, including overt hepatic encephalopathy (HE), ascites, and bacterial infection. Overt HE was diagnosed and graded using the West Haven Criteria after excluding other causes of neuropsychological disorders through history taking and neurological examination. Ascites was detected using abdominal imaging or other clinical findings. Bacterial infection was diagnosed according to the criteria as previously published^15,16^.

The study protocol and patient consent form were approved by the Institutional Review Board, Faculty of Medicine, Chulalongkorn University (IRB No. 330/59). All participants or their legal representatives provided written informed consent to participate in the study. The study protocol adhered to the Helsinki Declaration's ethical standards and the Good Clinical Practice recommendations.

### Laboratory measurement

Baseline laboratory parameters, arterial lactate, and ammonia levels were evaluated. Arterial ammonia samples were obtained at admission. Samples were collected in a heparinized syringe and quickly transported on ice to the central laboratory for measurement.

### Management protocol

All patients were managed in accordance with local protocols. Broad-spectrum antibiotics were given to treat infection in accordance with each hospital’s guidelines, following blood collection and a septic workup. Patients with hepatitis B virus infection were immediately treated with anti-viral therapy. Patients using hepatotoxic medications or alcohol consumption were required to discontinue. Intravenous crystalloids, as well as human albumin were administered for fluid resuscitation and terlipressin, if treating hepatorenal syndrome. Patients with HE were treated with lactulose and rifaximin. Organ support was administered as needed with renal replacement therapy for renal failure, intubation and mechanical ventilation for respiratory failure, and vasopressors for circulatory failure.

### Statistical analysis

Categorical variables were presented as numbers and percentages and compared between groups using the Pearson Chi-square or Fisher's exact test. Continuous variables were analyzed for normality using the Shapiro–Wilk test. Normally distributed data were expressed as mean (standard deviation) with group means comparisons using the independent t-test, while non-normally distributed data were expressed as median (interquartile range) with group comparison testing using the Mann–Whitney U test. Multivariate logistic regression was done to assess the association with LRCs at baseline. Cox regression analyses identified potential factors associated with 30-day mortality. The Kaplan–Meier graph and survival predictions were compared using the Log-rank test. Area under the receiver operating characteristics (AUROC), and Youden’s index were analyzed to identify the optimal ammonia cutoffs for 30-day mortality. Comparison of AUROC was done by the Medcalc Software (Belgium) using Delong’s technique. Statistical analysis was performed using the Statistical Package for the Social Sciences (SPSS) software, Version 23 (IBM Corporation, Armonk, NY, USA). Two-sided *p*-values less than 0.05 were considered statistically significant.

## Results

### Patient baseline characteristics

A total of 3871 patients with ACLF were screened. Of these, 701 patients with ACLF were enrolled. The most common reason for exclusion was a lack of ammonia presence. Baseline characteristics of patients who were included and excluded were shown in the Supplementary Table 1. Patients who were excluded had higher rates of LRCs and alanine aminotransferase (ALT), but a lower proportion of ACLF grade II-III, total bilirubin (TB), INR, creatinine, and lactate levels than those who were included. In patients who were enrolled, most of the patients were male (85.6%, n = 600), with a median age of 43 (IQR: 37–52) years. The most common cause of chronic liver disease or cirrhosis was alcohol-related liver disease (84.2%, n = 590), followed by hepatitis B virus infection (15.8%, n = 111). The median Model for End-Stage Liver Disease (MELD), Sequential Organ Failure Assessment (SOFA), and AARC scores were 29.0 (IQR: 24.3–35.0), 8 (IQR: 6–10) and 9 (IQR: 8–11), respectively. At baseline, 127 patients (18.1%) had ACLF grade I, 364 (51.9%) had ACLF grade II, and 210 (30.0%) had ACLF grade III. In addition, 427 patients (60.9%) had at least one LRC, 346 (49.4%) had ascites, 276 (39.4%) had overt HE, and 245 (35.0%) had bacterial infections at admission. Baseline patient characteristics according to each LRC, overt HE, ascites, and bacterial infection were analyzed and shown in the Supplementary Table 2. ACLF with overt HE, ascites, or bacterial infection were sicker, as evidenced by higher MELD score than those without these complications.

### Ammonia level, severity of ACLF, and liver-related complications at admission

Ammonia levels increased with the severity of ACLF with mean values of 117.2 ± 91.6, 131.4 ± 74.2, and 161.5 ± 113.2 µmol/L (p < 0.001) in ACLF grades I, II, and III, respectively. Baseline demographics and characteristics of LRC presence are shown in Table 1. Patients with LRCs had a larger proportion of ACLF grade III as well as higher white blood cell counts (WBC), ALT, INR, serum lactate, MELD, and AARC scores. Ammonia levels were significantly higher in patients who had at least one LRCs than those without LRCs [133 (IQR: 98.0–194.7) vs. 69 (IQR: 36.1–141.0) µmol/L, p < 0.001]. Baseline ammonia levels were significantly higher in ACLF patients with ascites [122 (IQR: 71.2–198.8) vs. 107 (IQR: 67–160.5) µmol/L, p < 0.001] and overt HE [136.1 (IQR: 71.2–198.8) vs. 111 (IQR: 67–160.5) µmol/L, p = 0.02] than those without these complications. Patients with bacterial infections showed a non-significant difference compared to those without bacterial infections [125 (IQR: 75.6–180.1) vs. 114 (IQR: 64–171) µmol/L, p = 0.09].

**Table 1:** Baseline patient characteristics based on the presence of liver-related complications.

	No LRCs (n = 274)	LRCs (n = 427)	p-value
Age, years	43 (36–51)	45 (37–53)	0.32
Female, n (%)	44 (16.1%)	57 (13.3%)	0.32
Etiology, n (%)			
ARLD	227 (82.8%)	363 (85%)	0.44
HBV	47 (17.2%)	64 (15.0%)	
ACLF grade			
I	71 (25.9%)	54 (12.6%)	< 0.001
II	153 (55.8%)	208 (48.7%)	0.09
III	50 (18.3%)	165 (38.7%)	< 0.001
Hemoglobin, g/dL	10.5 (8.8–12.2)	10.2 (8.7–11.8)	0.22
White cell count,cells/µL	11,400 (6,460–17,140)	12,150 (8,700–18,600)	0.008
Sodium, mmol/L	133 (128–136)	131 (126–136)	0.01
Total bilirubin, mg/dL	19.8 (13.3–27.2)	21.8 (13.5–28.4)	0.11
ALT, IU/L	46.0 (23.0–93.3)	59 (36–115)	< 0.001
Albumin, g/dL	2.3 (1.8–2.9)	2.2 (1.8–2.7)	0.17
INR	2.0 (1.7–2.6)	2.4 (1.9–3.1)	< 0.001
Creatinine, mg/dL	1.0 (0.7–1.8)	1.2 (0.7–2.0)	0.39
Ammonia, µmol/L	69.0 (36.1–141.0)	133 (98–194.7)	< 0.001
Lactate, mmol/L	1.6 (1.3–2.2)	1.8 (1.4–2.8)	< 0.001
MELD	26.4 (23–32.9)	30.5 (25.3–36.3)	< 0.001
SOFA score	8 (6–10)	9 (7–10.3)	0.20
AARC score	9 (7–10)	10 (9–12)	< 0.001

AARC; ACLF Research Consortium, ACLF; acute-on-chronic liver failure, ALT; alanine aminotransferase, ARLD; alcohol-related liver disease, LRCs; liver-related complications, MELD; Model for End-Stage Liver Disease score, SOFA; Sequential Organ Failure Assessment score.

Results from the univariate analysis revealed that total WBC, TB, INR, and ammonia levels were associated with LRCs. Multivariate analysis showed that ammonia levels (OR = 1.011, 95%CI 1.009–1.014, p < 0.001) was independently associated with LRCs (Table 2).

**Table 2:** Factors related to liver-related complications at admission.

Factor	Univariate analysis			Multivariate analysis		
	OR	95% CI	p-value	OR	95% CI	p-value
Age	1.007	0.994–1.021	0.28			
Female	0.805	0.526–1.233	0.32			
Hemoglobin	0.964	0.902–1.031	0.28			
WBC	1.023	1.006–1.039	0.01	1.005	0.990–1.020	0.50
Sodium	0.980	0.961–1.000	0.50			
TB	1.014	0.998–1.030	0.08	1.002	0.985–1.020	0.79
ALT	1.000	1.000–1.001	0.54			
Albumin	0.901	0.729–1.113	0.33			
INR	1.371	1.159–1.623	< 0.001	1.183	0.997–1.403	0.05
Creatinine	0.981	0.885–1.087	0.71			
Ammonia	1.012	1.009–1.015	< 0.001	1.011	1.009–1.014	< 0.001

ALT; alanine aminotransferase, TB; total bilirubin, WBC; white blood cell count.

WBC, TB, INR, and ammonia were included in the multivariate analysis.

### Factor associated with 30-day mortality

Overall, the 30-day mortality rate was 49.9% (n = 350). ACLF patients who died within 30 days after hospitalization were older and had a proportional higher incidence of alcohol-related liver disease, baseline LRCs, and ACLF grade III. Higher baseline MELD, SOFA, and AARC scores were also reported compared to patients who survived the 30-day period. Baseline ammonia (p < 0.001) and lactate (p < 0.001) was significantly lower in survivors compared to non-survivors (Supplementary Table 3).

Results from the univariate Cox regression analysis found that older age, baseline hemoglobin, WBC, TB, ALT, INR, creatinine, ammonia levels, MELD, and AARC score were associated with 30-day mortality. In multivariate analysis, model 1 revealed that older age (HR = 1.022, 95% CI 1.013–1.032, p < 0.001), lower hemoglobin (HR = 0.941, 95% CI 0.894–0.989, p = 0.02), high INR (HR = 1.219, 95% CI 1.126–1.320, p < 0.001), serum creatinine (HR = 1.154, 95% CI 1.089–1.224, p < 0.001), arterial ammonia (HR = 1.002, 95% CI 1.001–1.003, p < 0.001) and ALT (HR = 1.000, 95%CI 1.000–1.001, p < 0.001) were independent predictors of 30-day mortality in ACLF patients (Table3). Ammonia levels were independent predictors of 30-day mortality when adjusting for MELD score (Model 2) and AARC score (Model 3). Baseline ammonia levels predicted 30-day mortality with an AUROC of 0.58 (95% CI 0.53–0.62, p = 0.001). However, the accuracy of ammonia was less than baseline AARC score (p < 0.001) in predicting 30-day mortality (AUROC = 0.68, 95%CI 0.64–0.72).

**Table 3:** Predictor for 30-day mortality in ACLF patients.

Factor	Univariate analysis			Multivariate analysis Model 1			Multivariate analysis Model 2			Multivariate analysis Model 3		
	HR	95% CI	p-value	HR	95% CI	p-value	HR	95% CI	p-value	HR	95% CI	p-value
Age	1.016	1.006–1.025	0.001	1.022	1.013–1.032	< 0.001	1.021	1.011–1.030	< 0.001	1.019	1.009–1.028	< 0.001
Female	1.042	0.775–1.403	0.14									
Hemoglobin	0.949	0.905–0.994	0.03	0.941	0.894–0.989	0.02	0.947	0.899–0.998	0.04	0.949	0.900–1.000	0.05
WBC	1.007	1.000–1.014	0.07	1.001	0.999–1.012	0.75	1.004	0.995–1.013	0.35	0.999	0.991–1.008	0.84
Sodium	0.996	0.983–1.010	0.62									
TB	1.014	1.003–1.025	0.01	1.012	0.994–1.023	0.07						
ALT	1.000	1.000–1.001	0.008	1.000	1.000–1.001	< 0.001	1.000	1.000–1.001	< 0.001	1.000	1.000–1.001	0.03
Albumin	1.022	0.884–1.181	0.77									
INR	1.213	1.131–1.301	< 0.001	1.219	1.126–1.320	< 0.001						
Creatinine	1.138	1.077–1.202	< 0.001	1.154	1.089–1.224	< 0.001						
Ammonia	1.002	1.001–1.003	< 0.001	1.002	1.001–1.003	< 0.001	1.001	1.000–1.002	0.03	1.001	1.000–1.002	0.04
MELD score	1.053	1.039–1.067	< 0.001				1.052	1.037–1.066	< 0.001			
AARC score	1.232	1.172–1.295	< 0.001							1.219	1.159–1.283	< 0.001

ALT; alanine aminotransferase, TB; total bilirubin, WBC; white blood cell count.

Model 1 included age, hemoglobin, WBC, TB, ALT, INR, creatinine, and ammonia.

Model 2 included age, hemoglobin, WBC, ALT, ammonia, and MELD score.

Model 3 included age, hemoglobin, WBC, ALT, ammonia, and AARC score.

**Figure 1:**
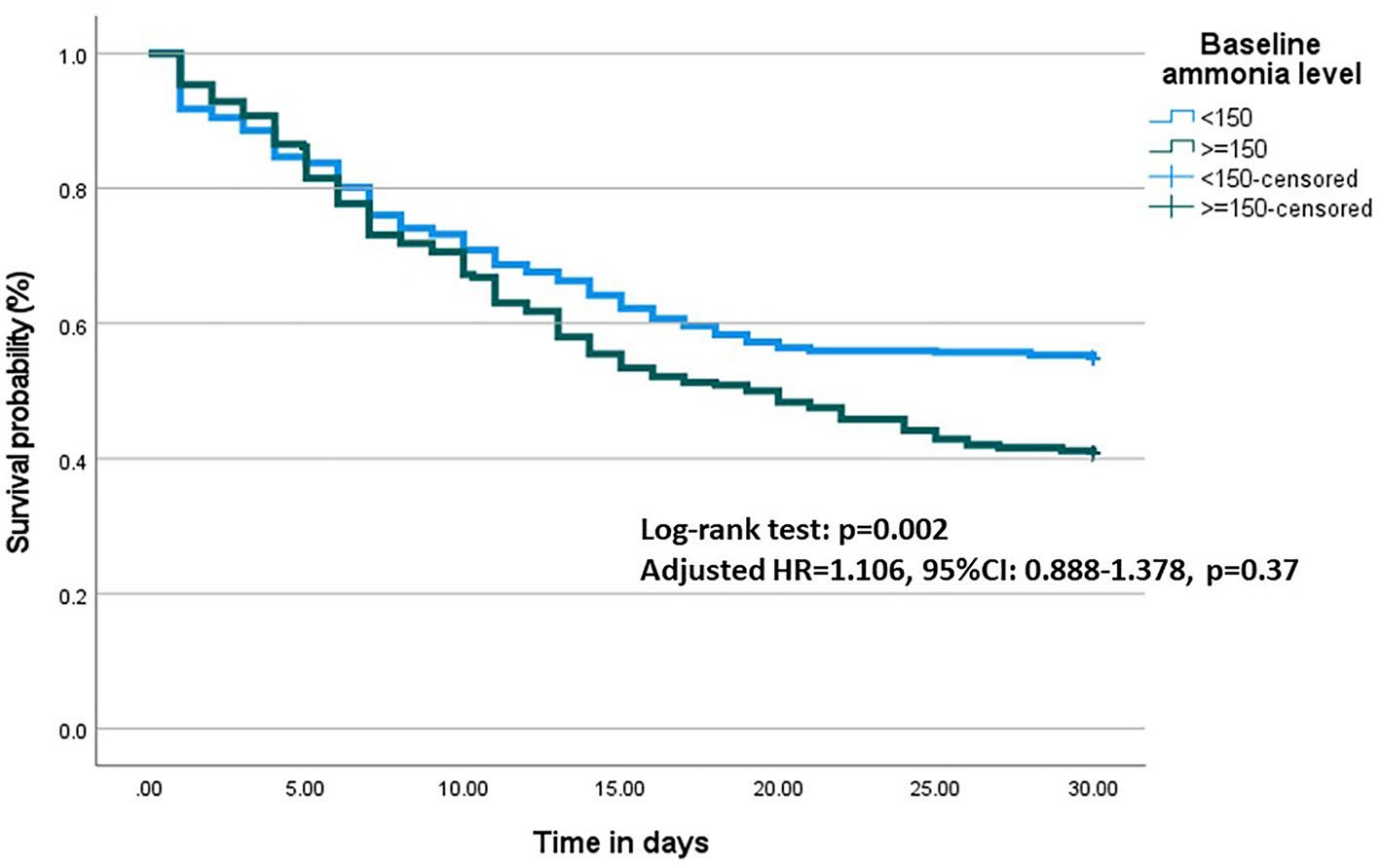
The Kaplan–Meier survival analysis stratified by ammonia level.

By maximizing Youden’s index, the optimal cutoff of baseline ammonia to predict 30-day mortality was ≥ 150 µmol/L with 40.3% sensitivity, 72.4% specificity, 59.2% positive predictive value, 54.9% negative predictive value, and an AUROC of 0.56 (95%CI 0.52–0.61, p = 0.004). The Kaplan–Meier survival analysis stratified by ammonia level is shown in Fig. 1. Patients with baseline ammonia < 150 µmol/L had a higher 30-day survival rate than those with ammonia ≥ 150 µmol/L (log-rank test; p = 0.002). However, ammonia ≥ 150 µmol/L was not an independent predictor for 30-day mortality after adjusting with MELD score (adjusted HR = 1.106, 95%CI 0.888–1.378, p = 0.37).

### Ammonia as a predictor of liver-related complications development

At the time of admission, 274 out of 701 patients (39.1%) did not have any LRCs. During the 30-day follow-up, 63 patients (23%) developed at least one LRC, 23 (8.4%) developed ascites, 13 (4.7%) had bacterial infections, and 21 got overt HE (7.7%). Patients who developed LRCs exhibited higher arterial ammonia than those without LRC development (124.6 ± 108.7 vs. 83.4 ± 64.5 µmol/L, p = 0.04). Ammonia levels were also significantly higher in those with ascites or overt HE compared to those without these complications, while ammonia levels were not statistically different in patients with or without the development of bacterial infection over 30 days. Univariate analysis revealed that only baseline ammonia (OR = 1.006, 95% CI 1.003–1.010, p < 0.001) predicted the development of LRCs during the 30-day follow-up period. (Supplementary Table 4). Patients with LRCs development during 30 days had a higher 30-day mortality rate than those without LRCs development (42.7% vs. 28.6%, p = 0.04).

## Discussion

The current study investigated the association between arterial ammonia levels and LRCs in patients with ACLF. We also assessed the potential role of arterial ammonia in predicting mortality and development of LRCs during the 30 days after hospitalization. Our main finding showed that baseline ammonia levels are associated with LRC, particularly overt HE, and ascites. In addition, arterial ammonia is a potential predictor of 30-day mortality and LRC development. The study findings have highlighted the additional role of ammonia in detecting and predicting poor clinical outcomes other than HE in ACLF patients.

Hyperammonemia is traditionally described as the central contributor of HE development. It is also associated with non-neurological organ injury. Ammonia exerts multiorgan dysfunction by promoting hepatocyte death, activation of hepatic stellate cells, and producing neutrophil phagocytic impairment, resulting in liver inflammation, fibrosis, portal hypertension, and increased risk for infection^17–19^. Tranah et al*.* demonstrated that ammonia independently predicts 1-year mortality and LRCs such as bacterial infection, variceal bleeding, ascites, and overt HE in clinically stable outpatients with cirrhosis^11^. In addition, ammonia outperforms Child–Pugh and MELD scores in predicting complications. Balcar et al. recently showed that ammonia is linked to liver-related death, liver dysfunction, and portal hypertension severity in clinically stable outpatients with advanced chronic liver disease^10^. In sicker patients, such as those with ACLF, ammonia is associated with mortality^8,12,20,21^, severity of ACLF^12^, and an increased risk of organ failure^12,21^. Consistent with the current study’s findings, ammonia is independently associated with the severity of ACLF and 30-day mortality. To our knowledge, the study presents new evidence that ammonia is also independently related to the presence of LRCs, not only overt HE but also ascites in patients with ACLF. Although the study did not demonstrate the association between bacterial infection and high ammonia levels. This finding should be considered in the context that seventy percent (n = 172/245) of patients with bacterial infection had more than one liver-related complication. This could potentially impact the relationship between this complication and ammonia level. Further analysis in ACLF without baseline LRCs found that ammonia level predicts the new onset of LRC development within 30 days after admission. These findings support the additional prognostic role of ammonia in detecting and predicting complications in ACLF patients. However, the current study found that baseline ammonia levels ≥ 150 µmol/L was associated with 30-day mortality, but the AUROC was fair and had low sensitivity.

There is an unmet need for a universal definition of ACLF. Three main definitions from different consortiums around the world have been proposed from the APASL AARC, European Association for the Study of the Liver-Chronic Liver Failure (EASL-CLIF) consortium^22^, and the North American Consortium for the Study of End-Stage Liver Disease (NACSELD)^23^. Organ failure is one of the key differences among the three guidelines. APASL AARC requires hepatic failures, EASL-CLIF requires a combination of hepatic and extrahepatic organ failure, and NACSELD needs at least two extrahepatic organ failures. ACLF is characterized by acute hepatic insult presenting as jaundice and coagulopathy, followed by ascites and/or encephalopathy within 4 weeks in patients with prior chronic liver disease or cirrhosis by APASL guidelines^14^. The feature of acute decompensation indicates significant hepatic deterioration, supporting the importance of hepatic failure in these criteria. ACLF precipitated by hepatic insults is distinct from that caused by extrahepatic insults^24^. The presence of LRC in ACLF may indicate prominent portal hypertension. Development of LRCs is associated with high 6-week mortality and poor outcomes in patients with ACLF^25^. In addition, hepatic decompensation indicates poor reversibility of ACLF syndrome^26^. According to this study's findings, ammonia may guide physicians in managing these complications.

There are some limitations in this study. First, there could be discrepancies in technical concerns with ammonia testing among centers, even though investigators checked the test’s reliability before entering data. Strict sample handling and processing requirements were the possible explanations for the differences in ammonia levels among testing sites^27^. Ammonia level is affected by various factors such as the centrifugation process, blood drawing tourniquet technique, transportation, and hemolysis. Second, the study only enrolled participants with alcohol-related liver disease and hepatitis B virus, since these are two common causes of chronic liver disease in the Asia–Pacific region. Third, variceal bleeding was not included in the list of liver-related complications due to the inaccessibility of this data in the majority of the hospitals. Fourth, there were a small number of patients without baseline LRCs who developed LRCs during the 30 days of follow-up, which might affect the result’s validity. In addition, the management of ACLF relied on the decision-making of physicians in each hospital, which could impact the outcome of patients. If the patients were discharged earlier than day 30, they were re-evaluated clinical information at the outpatient clinic on the 30th day after enrollment. Ascites was assessed using abdominal imaging or clinical findings according to the attending physician's decision at each hospital. Therefore, patients with grade 1 ascites might be overlooked if they were not evaluated by ultrasound.

In conclusion, baseline arterial ammonia levels demonstrated an association with 30-day mortality and LRCs, encompassing not only overt HE but also ascites in patients with ACLF.

### Supplementary Information

Supplementary Tables.

## Data Availability

The datasets used and/or analysed during the current study available from the corresponding author on reasonable request.
