# Supplementary material for: Ammonia is associated with liver-related complications and predicts mortality in acute-on-chronic liver failure patients
Source: Sci Rep. 2024 Mar 9;14:5796. doi: 10.1038/s41598-024-56401-x (PMC10924893; doi:10.1038/s41598-024-56401-x)
Supplement: Supplementary file 1 — Supplementary Tables. [file 41598_2024_56401_MOESM1_ESM.docx]

**Supplementary Table 1: Baseline patient characteristics of ACLF patients who were enrolled and excluded from the study.**

|  | **Patients who were excluded (n=3,170)** | **Patients who were enrolled (n=701)** | **p-value** |
| --- | --- | --- | --- |
| Age, years | 43.0 (36.0-52.0) | 43.0 (37.0-52.0) | 0.24 |
| Female, n (%) | 374 (13.9%) | 101 (14.4%) | 0.73 |
| Aetiology, n (%)   - ARLD | 2,615 (82.5%) | 590 (84.2%) | 0.60 |
| ACLF grade |  |  |  |
| - II-III | 2,064 (76.8%) | 530 (82.2%) | 0.003 |
| Hemoglobin, g/dL | 10.4 (8.9-11.9) | 10.0 (8.8-12.0) | 0.58 |
| White cell count, cells/µL | 12,000 (7,800-18,500) | 11,725 (7,900-17,800) | 0.45 |
| Sodium, mmol/L | 132 (127-136) | 132 (127-136) | 0.96 |
| Total bilirubin, mg/dL | 18.0 (10.7-25.6) | 20.9 (13.4-28.0) | < 0.001 |
| ALT, IU/L | 61.0 (36.0-134.0) | 54.4 (30.0-104.0) | < 0.001 |
| Albumin, g/dL | 2.30 (1.80-2.70) | 2.20 (1.80-2.80) | 0.63 |
| INR | 2.15 (1.78-2.73) | 2.16 (1.83-2.91) | 0.04 |
| Creatinine, mg/dL | 1.00 (0.66-1.80) | 1.10 (0.70-1.93) | 0.01 |
| Lactate | 1.70 (1.20-2.50) | 1.70 (1.30-2.60) | 0.02 |
| MELD | 27.8 (24.0-33.6) | 29.0 (24.3-35.0) | 0.05 |
| SOFA score | 9.0 (7.0-10.0) | 8.0 (6.0-10.0) | 0.15 |
| AARC score | 9.0 (8.0-11.0) | 9.0 (8.0-11.0) | 0.21 |
| Liver-related complication | 2,029 (75.5%) | 427 (60.9%) | < 0.001 |

AARC; ACLF Research Consortium, ACLF; acute-on-chronic liver failure, ALT; alanine aminotransferase, ARLD; alcohol-related liver disease, MELD; Model for End-Stage Liver Disease score, SOFA; Sequential Organ Failure Assessment score

**Supplementary Table 2: Baseline patient characteristics of ACLF patients according to overt HE, ascites, and bacterial infection**

|  | **Overt HE** | | | **Ascites** | | | **Bacterial infection** | | |
| --- | --- | --- | --- | --- | --- | --- | --- | --- | --- |
|  | **No (n=425)** | **Yes**  **(n=276)** | **p-value** | **No**  **(n =355)** | **Yes**  **(n=346)** | **p-value** | **No**  **(n=456)** | **Yes**  **(n=245)** | **p-value** |
| Age, years | 43.0 (36.0-50.0) | 46.0 (37.0-57.0) | 0.002 | 43.0 (37.0-52.0) | 43.5 (36-52.3) | 0.90 | 42.5 (36.0-51.0) | 46.0 (37.0-55.0) | 0.02 |
| Female, n (%) | 53 (12.5%) | 48 (17.4%) | 0.07 | 65 (18.3%) | 36 (10.4%) | 0.003 | 62 (13.6%) | 39 (15.9%) | 0.41 |
| ARLD | 377 (88.7%) | 213 (77.2%) | <0.001 | 302 (85.1%) | 288 (83.2%) | 0.51 | 380 (83.3%) | 210 (85.7%) | 0.41 |
| ACLF grade II-III, n (%) | 318 (78.1%) | 212 (89.1%) | <0.001 | 253 (8.01%) | 277 (84.2%) | 0.17 | 335 (81.7%) | 195 (83.0%) | 0.69 |
| Hemoglobin, g/dL | 10.4 (8.8-12.0) | 10.2(8.0-19.3) | 0.83 | 10.4 (8.8-12.1) | 10.3 (8.7-12.0) | 0.66 | 10.4 (8.8-12.1) | 10.1 (8.6-11.7) | 0.16 |
| White cell count, cells /µL | 11,700 (7,712-17,105) | 11,800 (7,975-19,275) | 0.44 | 11,700 (7,100-19,365) | 11,795 (8,400-16,500) | 0.96 | 12,100 (7,750-18,300) | 11,400 (8,050-16,900) | 0.41 |
| Sodium, mmol/L | 131.8 (127.5-136.0) | 132.0 (127.0-136.0) | 0.54 | 132.2 (128.0-136.0) | 131.0 (126.2-135.7) | 0.04 | 132.0 (128.0-136.0) | 131.0 (126.0-135.4) | 0.02 |
| Total bilirubin, mg/dL | 20.1 (12.2-27.8) | 22.2 (15.1-28.2) | 0.09 | 20.0 (12.9-27.8) | 21.8 (14.2-28.1) | 0.15 | 20.9 (13.5-28.2) | 21.4 (13.3-27.8) | 0.41 |
| ALT, IU/L | 53 (30-103) | 58 (31.3-111.5) | 0.50 | 51.0 (28.0-100.0) | 57.5 (35.0-109.0) | 0.04 | 54.0 (30.3-101.8) | 55.0 (30.0-112.0) | 0.78 |
| Albumin, g/dL | 2.2 (1.8-2.7) | 2.4 (1.9-2.9) | 0.002 | 2.2 (1.8-2.8) | 2.2 (1.8-2.7) | 0.44 | 2.3 (1.8-2.8) | 2.2 (1.8-2.7) | 0.34 |
| INR | 2.1 (1.8-2.8) | 2.2 (1.9-3.1) | 0.06 | 2.1 (1.8-2.8) | 2.3 (1.9-3.0) | 0.02 | 2.1 (1.8-2.9) | 2.3 (1.9-3.0) | 0.11 |
| Creatinine, mg/dL | 1.1 (0.7-1.8) | 1.2 (0.8-2.1) | 0.04 | 1.1 (0.7-1.9) | 1.2 (0.7-2.0) | 0.39 | 1.0 (0.7-1.6) | 1.3 (0.8-2.6) | <0.001 |
| Ammonia, µmol/L | 111.0 (67.0-160.5) | 136.1 (71.2-198.8) | 0.02 | 107.0 (67-160.5) | 122 (71.2-198.8) | <0.001 | 114.0 (64.0-171.1) | 125.0 (75.6-180.1) | 0.09 |
| Lactate, mmol/L | 1.7 (1.3-2.5) | 1.9 (1.4-3.2) | 0.002 | 1.7 (1.3-2.5) | 1.8 (1.4-2.8) | 0.01 | 1.7 (1.3-2.6) | 1.9 (1.4-2.8) | 0.02 |
| MELD | 27.7 (23.7-34.0) | 31.6 (25.8-37.5) | < 0.001 | 27.6 (23.5-34.3) | 30.5 (25.6-36.0) | <0.001 | 27.9 (23.7-33.6) | 31.7 (25.8-37.8) | <0.001 |
| SOFA score | 8.0 (6.0-9.0) | 9.0 (7.0-11.0) | < 0.001 | 8.0 (6.0-10.0) | 9.0 (7.0-10.0) | 0.37 | 8.0 (6.0-11.0) | 8.0 (6.0-10.0) | 0.27 |
| AARC score | 9.0 (8.0-10.0) | 11.0 (9.0-12.0) | < 0.001 | 9.0 (8.0-10.0) | 10.0 (8.0-12.0) | <0.001 | 9.0 (8.0-11.0) | 10.0 (8.0-11.0) | 0.26 |

AARC; ACLF Research Consortium, ACLF; acute-on-chronic liver failure, ALT; alanine aminotransferase, ARLD; alcohol-related liver disease, MELD; Model for End-Stage Liver Disease score, SOFA; Sequential Organ Failure Assessment score

**Supplementary Table 3: Baseline patient characteristics of ACLF patients who survived and died during 30 days after admission.**

|  | **Survived (n=351)** | **Died (n=350)** | **p-value** |
| --- | --- | --- | --- |
| Age, years | 43 (36-50) | 45.0 (37.0-19.6) | 0.02 |
| Female, n (%) | 50 (14.2%) | 51 (14.6%) | 0.90 |
| Aetiology, n (%)   - ARLD - HBV | 276 (78.6%)  75 (21.4%) | 314 (89.7%)  36 (10.3%) | <0.001 |
| ACLF grade |  |  |  |
| - I | 117 (23.4%) | 64 (12.8%) | <0.001 |
| - II | 307 (61.3%) | 213 (42.6%) | <0.001 |
| - III | 77 (15.3%) | 223 (44.6%) | <0.001 |
| Hemoglobin, g/dL | 10.3 ± 2.0 | 10.4 ± 2.4 | 0.57 |
| White cell count, cells/µL | 10,805 (6,793-16,500) | 13,100 (9,125-19,618) | <0.001 |
| Sodium, mmol/L | 132.1 (127.7-136.0) | 131.6 (126.1-136.0) | 0.28 |
| Total bilirubin, mg/dL | 19.8 (12.0-27.1) | 22.3 (15.1-29.0) | 0.005 |
| ALT, IU/L | 52.0 (26.0-92.8) | 60.0 (35.0-118.0) | 0.001 |
| Albumin, g/dL | 2.2 (1.8-2.8) | 2.2 (1.8-2.8) | 0.84 |
| INR | 2.1 (1.8-2.6) | 2.3 (1.9-3.3) | <0.001 |
| Creatinine, mg/dL | 1.0 (0.6-1.5) | 1.3 (0.8-2.4) | <0.001 |
| Ammonia, µmol/L | 106.0 (62.4-157.0) | 127.2 (78.0-191.3) | <0.001 |
| Lactate | 1.6 (1.3-2.2) | 1.9 (1.4-3.2) | <0.001 |
| MELD | 26.2 (23.5-31.8) | 31.5 (26.1-38.5) | <0.001 |
| SOFA score | 8.0 (6.0-9.0) | 9.0 (7.0-12.0) | <0.001 |
| AARC score | 9.2 (8.0-10.0) | 10.0 (9.0-12.0) | <0.001 |
| Liver-related complication | 185 (52.7%) | 242 (69.1%) | <0.001 |

AARC; ACLF Research Consortium, ACLF; acute-on-chronic liver failure, ALT; alanine aminotransferase, MELD; Model for End-Stage Liver Disease score, SOFA; Sequential Organ Failure Assessment score

**Supplement Table 4:** **Predictor of liver-related complications during 30 days in ACLF patients who did not have baseline LRCs.**

| Factor | **Univariate analysis** | | | **Multivariate analysis** | | |
| --- | --- | --- | --- | --- | --- | --- |
|  | **OR** | **95% CI** | **p-value** | **OR** | **95% CI** | **p-value** |
| Age | 0.986 | 0.962-1.011 | 0.269 |  |  |  |
| Female | 0.982 | 0.455-2.119 | 0.964 |  |  |  |
| Hemoglobin | 1.015 | 0.895-1.150 | 0.821 |  |  |  |
| White cell count | 0.995 | 0.963-1.028 | 0.758 |  |  |  |
| Sodium | 1.041 | 0.997-1.087 | 0.068 |  |  |  |
| Total bilirubin | 1.023 | 0.992-1.054 | 0.145 |  |  |  |
| ALT | 1.000 | 1.000-1.000 | 0.620 |  |  |  |
| Albumin | 1.242 | 0.865-1.785 | 0.240 |  |  |  |
| INR | 0.993 | 0.728-1.353 | 0.964 |  |  |  |
| Creatinine | 0.921 | 0.760-1.111 | 0.396 |  |  |  |
| Ammonia | 1.006 | 1.003-1.010 | <0.001 |  |  |  |
| MELD | 0.985 | 0.946-1.025 | 0.465 |  |  |  |

ALT; alanine aminotransferase, MELD; Model for End-Stage Liver Disease score
